# Target Attainment and Population Pharmacokinetics of Cefazolin in Patients with Invasive *Staphylococcus aureus* Infections: A Prospective Cohort Study

**DOI:** 10.3390/antibiotics13100928

**Published:** 2024-09-29

**Authors:** Severin Bausch, Sarah Dräger, Panteleimon Charitos-Fragkakis, Adrian Egli, Stephan Moser, Vladimira Hinic, Richard Kuehl, Stefano Bassetti, Martin Siegemund, Katharina M. Rentsch, Laura Hermann, Verena Schöning, Felix Hammann, Parham Sendi, Michael Osthoff

**Affiliations:** 1 Division of Internal Medicine, University Hospital Basel, 4031 Basel, Switzerland; 2 Department of Clinical Research, University of Basel, 4001 Basel, Switzerland; 3 Division of Clinical Bacteriology and Mycology, University Hospital Basel, 4031 Basel, Switzerland; 4 Institute of Medical Microbiology, University of Zurich, 8006 Zurich, Switzerland; 5 Division of Infectious Diseases and Hospital Epidemiology, University Hospital Basel, 4031 Basel, Switzerland; 6 Intensive Care Unit, Department of Acute Medicine, University Hospital Basel, 4031 Basel, Switzerland; 7 Department of Laboratory Medicine, University Hospital Basel, 4031 Basel, Switzerland; katharina.rentsch@usb.ch; 8 Division of Clinical Pharmacology & Toxicology, Department of Internal Medicine, University Hospital Bern, 3010 Bern, Switzerland; 9 Institute for Infectious Diseases, University of Bern, 3012 Bern, Switzerland; 10 Department of Internal Medicine, Cantonal Hospital Winterthur, 8400 Winterthur, Switzerland

**Keywords:** *Staphylococcus aureus*, cefazolin, therapeutic drug monitoring, target attainment, population pharmacokinetic model

## Abstract

This study aimed to determine cefazolin target attainment in patients with invasive *Staphylococcus aureus* (*S. aureus*) infections and to develop a population pharmacokinetic (PK) model. Adult patients with invasive *S. aureus* infections treated with cefazolin bolus infusions were included. Unbound and total trough and mid-dose cefazolin concentrations were measured, and strain-specific MICs were determined. The primary outcome was the proportion of patients attaining 100% *f*T_>MIC_ at all time points evaluated. A population PK model was developed, using non-linear mixed-effects modelling. Overall, 51 patients were included, with a total of 226 unbound and total cefazolin concentrations measured (mean: 4.4 per patient). The median daily dosage in patients with an estimated glomerular filtration rate of >60 mL/min/m^2^ was 8 g. The median age was 74 years (interquartile range (IQR) 57–82) and 26% were female. A history of chronic kidney disease and acute kidney injury were present in 10/51 (19.6%) and 6/51 (11.7%), respectively. Achievement of 100% *f*T_>MIC_ occurred in 86% of the patients and decreased to 45% when a target of 100% *f*T_>4xMIC_ was evaluated. The mean unbound cefazolin fraction was 27.0% (standard deviation (SD) 13.4). Measured and estimated mean cefazolin trough concentrations differed significantly [13.1 mg/L (SD 23.5) vs. 7.4 mg/L (SD 7.9), *p* < 0.001]. In the population PK model, elevated estimated creatinine clearance and bolus instead of continuous application were covariates for target non-attainment. In conclusion, cefazolin target achievement was high, and the measurement of the unbound cefazolin concentration may be favored. The Monte Carlo simulations indicated that target attainment was significantly improved with continuous infusion.

## 1. Introduction

A key determinant of the efficacy of beta-lactam antibiotics is the proportion of the dosing interval (T) during which the unbound drug concentration (*f*) is above the minimum inhibitory concentration (MIC) of the pathogen (*f*T_>MIC_). Especially in patients with severe infections and in critically ill patients, the achievement of a pharmacodynamic target of 100% *f*T_>MIC_ or even 100% *f*T_>4xMIC_ may improve the clinical outcome [1,2,3,4]. However, this target is frequently not achieved or is exceeded due to the altered metabolism of these patients, i.e., augmented renal clearance, impaired renal function and hypoalbuminemia [5,6,7]. 

Cefazolin is a beta-lactam antibiotic used for the treatment of *Staphylococcus aureus* (*S. aureus*) infections and inhibits the bacterial cell wall synthesis [8,9]. It is a hydrophilic drug, almost entirely eliminated by the kidneys, and has a high protein binding of 80% [10,11]. Protein binding may be very different between patients [12], which may lead to a variable unbound cefazolin fraction and, consequently, to a variable unbound drug concentration, impacting the assessment of pharmacodynamic target attainment. Studies on the target attainment of cefazolin in patients with *S. aureus* infections are scarce, and other cefazolin pharmacokinetic/pharmacodynamic (PK/PD) studies focus on target attainment when used as peri-operative prophylaxis [13,14,15] or in obese patients [16]. 

Current dosing regimens do not take into account patient individual and dynamically changing parameters, which may be associated with suboptimal drug exposure [17]. Because sampling under clinical conditions may not always be according to optimal times, and measurements may be missed during routine work at the bedside, non-linear mixed effects modeling may be performed to gauge pharmacokinetic exposure. The resulting population pharmacokinetic models may reduce bias, handle sparse data more efficiently [18], incorporate patient-specific variables, and allow for the extrapolation and prediction of future treatments (e.g., personalized dosing) [19].

This study evaluated the pharmacological target attainment of unbound cefazolin in patients with invasive *S. aureus* infections and aimed to develop a population PK model to provide a patient-specific dosing recommendation.

## 2. Results

### 2.1. Patient Characteristics

Overall, 240 patients with complicated *S. aureus* infection were screened, and 51 patients, with a total of 226 unbound and total cefazolin concentration measurements, were included in this study (Supplement, Figure S1). The main exclusion criteria were treatment with another antibiotic, early transfer, or discharge and declined informed consent. The median age was 74 years (interquartile range (IQR) 57–82), and thirteen patients (26%) were female. Patient characteristics are summarized in Table 1. 

### 2.2. Pharmacological Data

The majority of patients (n = 47, 92%) received 6 g of cefazolin every 8 h, whereas only four patients (8%) received 2 g every 12 h, adjusted to their renal function. On study day 1, the median mid-dose unbound cefazolin concentration was 12.1 mg/L (IQR 6.0–23.1), and the median unbound trough concentration was 5.4 mg/L (IQR 2.1–11.4) (Table 2). Two patients had very high unbound trough drug concentrations of 128.9 mg/L and 87.4 mg/L, in the context of a markedly reduced estimated glomerular filtration rate (eGFR) of 10 and 20 mL/min/1.73 m^2^, respectively.

**Figure 1:**
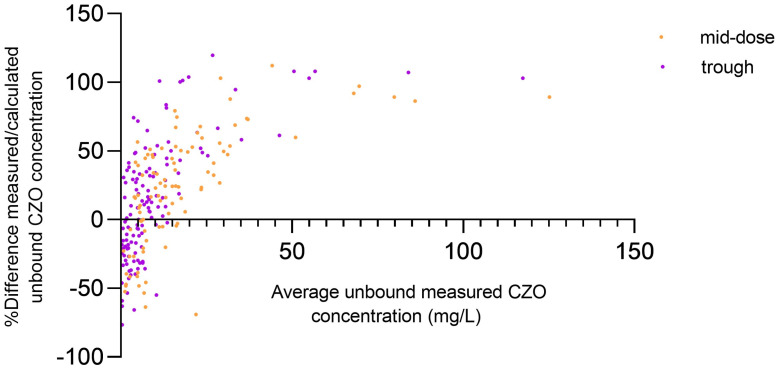
Bland–Altman plots of relative difference of measured/estimated unbound cefazolin concentration against the mean of average unbound measured cefazolin concentration. In mid-dose and trough concentrations, the bias was 23.5 and 14.78, and the 95% limits of agreement ranged from −57.7 to 104.7 and from −70.05 to 99.62, respectively. CZO: cefazolin.

The mean unbound cefazolin fraction was 27.0% (standard deviation (SD) 13.4; lower to upper value: 8.9% to 79.7%). The unbound fraction correlated significantly with serum albumin concentrations and eGFR (r = 0.58, *p* < 0.0001, and −0.42, *p* < 0.0001, respectively), determined at the same time as the cefazolin concentration (Supplement Figure S2), and was higher in critically ill patients compared to non-critically ill patients on the first study day [41.3% (SD 16.2) vs. 22.5% (SD 7.8), *p* = 0.003]. The disagreement between the measured and the estimated unbound cefazolin concentrations was significant [e.g., mean trough concentration: 13.1 mg/L (SD 23.5) vs. 7.4 mg/L (SD 7.9), *p* < 0.001, Figure 1] and more evident with lower albumin concentrations (Supplement Figure S3).

The median MIC of cefazolin was 1.0 mg/L (IQR 0.75–1.0) determined in 47/51 (92%) of S. aureus strains available. The highest measured MIC was 1.5 mg/L.

### 2.3. Pharmacological Target Attainment

The target of 100% *f*T_>MIC_ (strain-specific MIC) was achieved in 86% of the patients (n = 44/51), but only in 45% (n = 23/51) when a more ambitious target of 100% *f*T_>4xMIC_ was evaluated (Table 3). Although not statistically significant, critically ill patients more frequently achieved the higher target compared to non-critically ill patients (70% vs. 39%, *p* = 0.08). Similarly, a target of 100% *f*T_>MIC_ and 100% *f*T_>4xMIC_ was more frequently achieved by patients with an eGFR < 60 mL/min on admission or suffering from acute kidney injury (17/17 (100%) and 11/17 (65%), respectively), compared to patients with no evidence of acute or chronic renal injury (27/34 (79%) and 12/34 (35%), respectively, *p* = 0.08 for both comparisons). In the univariate logistic regression analysis, higher age, a higher Charlson Comorbidity Score, a higher SOFA score, and a lower eGFR were associated with the primary outcome (Supplement Table S1), all of which were not retained as significant in the stepwise multivariate analysis.

### 2.4. Excessive Cefazolin Concentrations and Potential Toxicity

In nine out of 51 patients (18%), the unbound trough concentration of cefazolin exceeded 20 mg/L (Table 3) at least once during the hospital stay and occurred more frequently in critically ill patients (56% vs. 9.8%, *p* = 0.003). In five patients, Glasgow coma scale (GCS) declined (1 to 11 points) during cefazolin treatment, two of which died within the follow-up period of 30 days. The median first cefazolin trough concentrations were 4.9 mg/L (IQR 1.8–9.3) and 48.8 mg/L (IQR 9.9–118.5), *p* = 0.01, in patients without and with GCS decline, respectively.

AKI occurred in six out of 51 patients (11.8%). The median first cefazolin trough concentrations were 4.9 mg/L (IQR 1.9–9.9) and 11.2 mg/L (IQR 8.9–97.8), *p* = 0.009, in patients not developing AKI and those who did, respectively.

### 2.5. Population Pharmacokinetics

All 51 patients were included in the population PK modeling. We developed a joint model to explain unbound and total concentrations simultaneously. We estimated one-, two-, and three-compartment structural base models with linear, zero-order, and Michaelis-Menten elimination. Our covariate modelling was guided by physiological plausibility and visual and numerical predictive diagnostics. As cefazolin is mainly eliminated renally and is highly protein-bound, eGFR according to Cockroft Gault (eGFR-GC), eGFR according to CKD-EPI (eGFR-CKD-EPI), weight, and albumin concentration were tested as covariates. We evaluated model performance using the goodness-of-fit diagnostic plots, visual predictive checks, the Aikake Information Criterion (AIC), the objective function value (OFV), and numerical predictive checks of competing models. The most important modeling steps and their respective OFVs are provided in Supplement Table S3.

A one-compartment model with the following relationship for total (C_total_), bound (C_bound_), and unbound (C_unbound_) concentrations described the data best:

$$\begin{array}{c} C_{bound}=\frac{B_{max\;}\times C_{unbound}}{kd+C_{unbound}}+NS\times C_{unbound}\\ C_{total}=C_{unbound}+C_{bound} \end{array}$$

where the maximum binding capacity (B_max_), a non-saturable constant (NS), and the dissociation constant (kd) parameterized albumin binding. The final parameter estimates for the model are given in Table S2, and the goodness-of-fit plots are shown in Figures S4 and S5.

**Figure 2:**
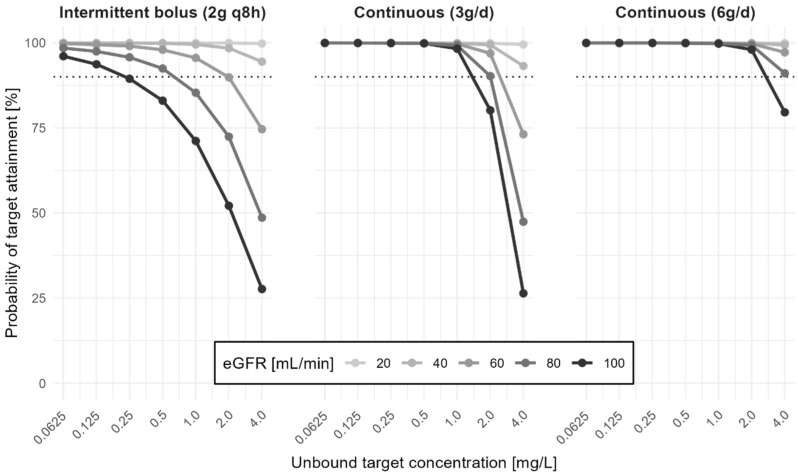
Influence of eGFR-CG on probability of target attainment (100% *f*T_>target concentration_) for different unbound target serum cefazolin concentrations for continuous (3 g/24 h and 6 g/24 h) and intermittent administration (2 g every 8 h).

Simulation of probability of target attainment showed a great impact of renal function and the administration route. Even though the daily dosage was only half for continuous compared to intermittent administration (3 g/d and 6 g/d, respectively), higher unbound serum concentrations and higher probability of target attainment were achieved with continuous infusion (Figure 2).

## 3. Discussion

In this prospective single-center study involving patients with invasive *S. aureus* infections, we demonstrated a high rate of pharmacodynamic target attainment (100% *f*T_>MIC_) throughout the study period, when unbound cefazolin concentrations were measured, and strain-specific MICs were used. We identified renal function and mode of administration as influencing covariates for target non-attainment in the population PK model.

In contrast to most previous studies, unbound cefazolin concentrations and strain-specific MICs of *S. aureus* were assessed, both non-automated, time-consuming, and resource-intensive processes with limited availability. However, these measurements may be crucial for the appropriate assessment of target attainment, as it may be markedly underestimated when an unbound cefazolin fraction of 20% and the epidemiological cutoff value (ECOFF) of 2 mg/L are used. The present study highlights that the estimation of the unbound cefazolin concentration is imprecise and may subsequently lead to inadequate dosing adjustments (particularly, to the administration of higher doses), especially in patients with low albumin and in critically ill patients [20]. These findings are in line with previous studies, revealing unbound cefazolin fractions ranging from 12% [21] to 40% [15]. In order to prevent the toxic adverse events of cefazolin, which is characterized by an increased relative pro-convulsive activity [22], the measurement of the unbound drug concentration should be favored, in particular in critically ill patients.

Data on cefazolin target attainment in patients with invasive *S. aureus* infections are very limited [17,23]. Comparability of target attainment in the present study with the few studies in adult patients with *S. aureus* infection is limited due to the use of different targets [100% *f*T_>MIC_ vs. 50% *f*T_>2xMIC_ [17]]. However, the median unbound cefazolin mid-dose concentrations were similar [17]. In the study conducted in critically ill children using intermittent or continuous infusions, target attainment was identical with the present study [23]. Both studies showed that target attainment decreases substantially when a more ambitious target of 100% *f*T_>4xMIC_ or the ECOFF is used [1,4,23]. More ambitious targets may be justified, as penetration into deep-seated sites of infection is required, and plasma concentrations may only act as surrogate parameters for tissue concentrations. However, a general increase in the dosage may not be appropriate given the heterogeneity of underlying organ dysfunctions.

Estimated GFR-CG was found to be a significant covariate for target (non)-attainment in the population PK model, which is in line with the results of previous studies [17,21,23,24,25]. Consequently, patients are at risk of inappropriate drug exposure if they have either an impaired renal function or augmented renal clearance [17,23]. In contrast to prior studies, albumin or weight were also identified as additional covariates in the population PK model, albeit at a smaller scale [16,23,25]. This may be explained by the small differences in weight, BMI, and albumin observed within the patients of our study cohort.

In the population PK model, cefazolin target attainment was also dependent on its mode of administration, favoring continuous infusion. According to it, a decrease of 20% of the total daily drug dosage is required for identical target attainment when cefazolin is administered as continuous infusion instead of intermittent bolus infusion, which is in line with a population PK model in children [23]. This finding may be particularly relevant for patients with augmented renal clearance to achieve the recommended pharmacodynamic targets. Of note, PK studies including total but not unbound drug concentrations may overestimate the total daily dose by underestimating the unbound fraction [24]. However, as current dosing guidelines cannot consider all these variables, and especially their dynamic changes, the implementation of population PK models and model-informed precision dosing in clinical practice may be needed to enable the next step towards personalized antibiotic dosing. This might be particularly relevant and recommended for the following subgroups: patients with the extremes of renal function (augmented vs. severely impaired renal function), patients on continuous cefazolin infusion, critically ill patients, and probably patients with difficult to treat infections where a more ambitious target (e.g., because of the limited cefazolin penetration) should be achieved.

This study has several limitations. Conclusions about target attainment in patients with renal replacement therapy cannot be drawn. The majority of patients were male, limiting the generalizability of the results. The MIC distribution of the *S. aureus* strains was relatively narrow and included only strains from a specific geographical area. Furthermore, given the small sample size, this study was not powered to assess clinical outcome variables (e.g., mortality).

## 4. Materials and Methods

### 4.1. Study Design and Setting

This single-center, prospective, observational cohort, and PK/PD study was conducted at the University Hospital Basel, a 750-bed tertiary care hospital in Switzerland, between January 2020 and December 2021. It was approved by the Ethics Committee of Northwest and Central Switzerland (EKNZ Project-ID: 2019-02229) and was registered at www.clinicaltrials.gov (NCT04503252) (accessed on 12 August 2024). All patients provided written informed consent for participation in the study.

### 4.2. Patient Selection and Management

All patients aged ≥ 18 years old, who had confirmed invasive methicillin-susceptible *S. aureus* infection and received or were intended to receive cefazolin, were screened for eligibility. Invasive *S. aureus* infection was defined as (i) *S. aureus* bloodstream infection (BSI) with a positive follow-up blood culture with *S. aureus*, or (ii) the presence of a deep-seated or invasive infection (e.g., vertebral osteomyelitis, septic arthritis). Exclusion criteria included previous inclusion in this study within the last 30 days, hemodialysis, pregnancy, outpatient treatment, polymicrobial BSI, infection caused by methicillin-resistant *S. aureus*, and termination of cefazolin treatment within 48 h. The patients who were admitted to the intensive care unit (ICU) were defined as critically ill patients. Cefazolin (2 g) was administered as an intermittent bolus infusion over 30 min, every 8 h, or every 6 h (e.g., in case of infective endocarditis). The dosage was 2 g every 12 h and 2 g every 24 h if eGFR was 10–30 or less than 10 mL/min/1.73 m^2^, respectively. None of the patients included in this study required renal replacement therapy during the study period. Clinical, demographic, and laboratory data were prospectively collected from the electronic health record of the patient.

### 4.3. Identification of Staphylococcus aureus and MIC Determination

Standard techniques were used to identify *S. aureus* from blood cultures and other specimens and to perform susceptibility testing, including the Virtuo system for blood culture incubation (bioMérieux, Lyon, France) and the VITEK2 system (bioMérieux). Cefazolin MICs were determined by MIC test strips (Liofilchem Diagnostici, Roseto degli Abruzzi, Italy). *S. aureus* strains were considered susceptible to cefazolin if the MIC was ≤2 mg/L. In case of missing MIC values (n = 4), an MIC of 1.5 mg/L was used for further analyses, the highest measured MIC in the present study.

### 4.4. Plasma Sampling and Drug Assay

The same plasma sample was used to determine cefazolin, albumin, and creatinine concentrations (including eGFR calculated by the Chronic Kidney Disease Epidemiology Collaboration (eGFR-CKD-EPI) equation.

Cefazolin plasma samples were drawn on study day 1 (mid-dose (i.e., after 50% of the dosing interval) and trough concentration), 3 ± 1 day (mid-dose and trough), 7 ± 2 days (trough), and 14 ± 5 days (trough) (Supplement, Figure S6). Subsequently, samples were stored at −80 °C immediately after blood collection and centrifugation and subsequently analyzed in batches. A validated high-performance liquid chromatography–tandem mass spectrometry (HPLC–MS/MS) method using isotope dilution was used to measure total and unbound cefazolin plasma concentrations [26]. Prior to analysis, unbound plasma concentrations were determined using ultracentrifugation. Manual protein precipitation and an online turbo flow extraction step were used for sample preparation. After chromatography, the analyte and the deuterated internal standard were separated and fragmented by positive electrospray ionization, and three major fragments were detected. The lower limit of quantification was 0.5 mg/L for the determination of total and unbound cefazolin. The assay was linear, in the range of 0.5–100.0 mg/L for total and unbound cefazolin. The intra- and inter-assay precision experiments over the entire concentration range resulted in a coefficient of variation of ≤10% for total cefazolin and ≤8.1% for unbound cefazolin.

### 4.5. Outcome

The primary outcome was the proportion of patients with target attainment (100% *f*T_>MIC_) at all measured timepoints using the unbound cefazolin plasma concentration and the strain-specific MIC. Secondary outcome measures included target attainment of 50% *f*T_>MIC_ and 100% *f*T_>4xMIC_ or using the ECOFF of cefazolin (2 mg/L) instead of the MIC. Additional outcome measures included the following: (i) the association of target attainment with patient demographics and laboratory variables; (ii) the difference in target attainment if estimated unbound concentrations were used (20% unbound fraction according to the literature) [20]; and (iii) assessment of potentially toxic side effects. Target attainment was assessed by using the strain-specific MIC.

### 4.6. Assessment of Potential Toxicity

An unbound cefazolin trough concentration of >20 mg/L [corresponding to 10× ECOFF of the European Society of Clinical Microbiology and Infectious Diseases (EUCAST)] was defined as the threshold for a potentially toxic drug concentration [6]. Nephrotoxicity was evaluated using the AKI index [27]. To determine neurotoxicity, changes in the GCS, according to Imani et al. [28], and the occurrence of seizures were assessed. Potential toxicity was evaluated during cefazolin treatment.

### 4.7. Modeling

The study source documentation did not include dose administrations before inclusion in this study, but it recorded the start date of cefazolin treatment. Using this information and the inter-dose interval of the first study dose, we imputed missing doses and, where applicable, assumed steady-state conditions (at least five drug administrations). To account for blood concentrations measured in different inter-dose intervals, we considered inter-occasional variability (IOV). No blood concentrations below the lower limit of quantification were observed.

The laboratory and clinical parameters (serum creatinine, serum albumin, hemofiltration, and ICU admission) were determined with each visit/ concentration measurement. As these parameters did not change substantially across occasions, we decided not to include them as time-varying covariates and only considered the first measurement for each patient and occasion. In addition to the eGFR (CKD-EPI), we also chose to evaluate the effect of estimated Cockcroft–Gault (eGFR-CG) [28,29]. We also considered different residual error models. A non-parametric bootstrap (n = 1000) was performed to validate the final joint model. The influence of renal function on the probability of target attainment was analyzed by Monte Carlo simulations, defined as an unbound serum concentration that is 100% of the time above a specified threshold. Simulation parameters included 10,000 virtual patients (albumin of 24.0844 g/L, weight of 70 kg, eGFR-CG of 20, 40, 60, 80, and 100 mL/min) in steady state with intermittent bolus administration (infusion time of 30 min) of 2 g every eight hours (6 g/24 h) or continuous infusions of 3 g/24 h. The percentage of patients being 100% of the time above the defined serum target concentration of cefazolin was calculated. Population pharmacokinetic modeling was performed in Monolix (2023R1, Lixoft SAS, a Simulations Plus company, Lancaster, CA, USA).

### 4.8. Statistical Analysis

We calculated that at least 50 cases would be necessary to obtain reasonably robust predictions to achieve the primary outcome and include up to three covariates in the empirical analyses. Continuous variables were analyzed using means, medians, SD, and IQR. Categorical variables were summarized using frequencies and percentages. We used the Mann–Whitney U-test, the Chi square test, and Fisher’s exact test where appropriate. For the correlation and normality testing, Spearman-correlation and the Shapiro–Wilk test were used. The Wilcoxon signed-rank test was used for the pairwise comparison of measured and estimated cefazolin concentrations.

Associations between patient variables and target attainment were analyzed using multivariable stepwise logistic regression models that included potentially confounding variables with a univariate *p* value of less than 0.1. Statistical significance was defined as a two-sided *p* value less than 0.05. All analyses and figures were performed using SPSS Version 28 (IBM SPSS Statistics for Windows. Armonk, NY, USA) and GraphPad (version 9; GraphPad Software, San Diego, CA, USA).

## 5. Conclusions

In conclusion, the pharmacodynamic target of 100% *f*T_>MIC_ was frequently attained in patients treated with cefazolin for invasive *S. aureus* infections when unbound cefazolin concentrations and strain-specific MICs were considered. However, this was not the case if a more ambitious target of 100%*f*T_>4xMIC_ was used, which may be relevant for critically ill patients. The estimation of the unbound cefazolin concentration is imprecise and may negatively impact subsequent dosing. In the population PK model, renal function and the mode of administration favoring continuous infusion were identified as important covariates for target attainment. Future strategies may consider population PK models to personalize antibiotic dosing.

## Figures and Tables

**Table 1:** Patient characteristics of the study population (51 patients).

Variable	N (%) or Median (IQR)
Female	13 (25.5)
Age (years)	74.1 (56.6–81.8)
BMI (kg/m^2^)	24.5 (21.8–29.6)
Weight (kg)	73 (67–93)
**Comorbidities**	
Cardiovascular disease	24 (47.1)
Chronic lung disease	11 (21.6)
Diabetes mellitus	11 (21.6)
Heart failure (NYHA II–IV)	11 (21.6)
Chronic kidney disease, stage G3	5 (9.8)
Chronic kidney disease, stage G4	5 (9.8)
Malignancies	5 (9.8)
IV drug use	5 (9.8)
Liver cirrhosis	3 (5.9)
Charlson comorbidity score	5 (2–7)
**Disease severity**	**at onset of infection**
Pitt bacteremia score	0 (0–1)
SOFA score	1 (1–3)
Bloodstream infection	39 (76.5)
**Laboratory results**	**at onset of infection**
C-reactive protein (mg/L)	157 (56–239)
White blood cells (106/L)	14.0 (10.8–17.2)
Creatinine (µmol/L)	81 (69–131)
eGFR (mL/min/1.7 m^2^)	76 (41–91)
Albumin (g/L)	29.0 (24.0–32.8)
**Focus of infection**	
Catheter or prosthetic material	13 (25.5)
Osteomyelitis or septic arthritis	10 (19.6)
Endocarditis	10 (19.6)
Skin and soft tissue	9 (17.6)
Respiratory tract	2 (3.9)
Intra-abdominal	1 (2.0)
Other	6 (11.8)
**Severity and clinical outcome**	
Vasoactive treatment	3 (5.9)
ICU admission	10 (19.6)
ICU LOS in days	5 (3–14)
Hospital length of stay in days	20 (16–38)
30-day mortality rate	3 (5.9)

Definitions: at onset of infection: the day the first invasive specimen tested positive for *Staphylococcus aureus*; at first study date: the day of first blood collection for cefazolin concentration measurement (study day d1). Data are presented as count (percentages) or median (interquartile range). BSI: blood stream infection; BMI: body mass index; NYHA: New York Heart Association; IV: intravenous; eGFR: estimated, glomerular filtration rate; SOFA: sequential organ failure assessment score; ICU: intensive care unit; LOS: length of stay.

**Table 2:** Cefazolin plasma concentrations according to the different timepoints during the study.

Cefazolin Concentration	Study Day 1		Study Day 3		Study Day 7	Study Day 14
	Mid-dose (n = 50)	Trough (n = 47)	Mid-dose (n = 46)	Trough (n = 42)	Trough (n = 28)	Trough (n = 13)
Total in mg/L, median (IQR)	51.6 (31.1–87.2)	26.4 (11.4–41.9)	56.0 (29.8–85.0)	32.2 (12.3–54.0)	22.7 (11.6–39.8)	24.5 (18.2–43.1)
Unbound fraction, %, mean (SD)	28.0 (12.3)	26.1 (12.3)	28.1 (12.4)	27.0 (15.7)	25.6 (15.2)	25 (14.6)
Measured unbound concentration in mg/L, median (IQR)	12.1 (6.0–23.1)	5.4 (2.1–11.4)	15.5 (5.7–29.5)	6.6 (2.2–13.8)	4.6 (2.0–14.3)	5.6 (3.7–16.2)
Estimated unbound concentration in mg/L *, median (IQR)	10.3 (6.2–17.4)	5.3 (2.3–8.4)	11.2 (6.0–17.0)	6.4 (2.5–10.8)	4.5 (2.3–8.0)	4.9 (3.6–8.6)

Data are presented as median (interquartile range) or means (standard deviation). * An unbound cefazolin fraction of 20% was used to estimate the unbound plasma concentration.

**Table 3:** Target attainment according to the study day, the target definition, and patient-specific minimum inhibitory concentration (MIC) or epidemiological cut-off value (ECOFF) used, n (%).

Pharmacological Target	Study Day 1		Study Day 3		Study Day 7		Study Day 14		Cumulative	
	Cefazolin MIC	ECOFF	Cefazolin MIC	ECOFF	Cefazolin MIC	ECOFF	Cefazolin MIC	ECOFF	Cefazolin MIC	ECOFF
≥50% ƒT_>MIC_, n (%)	49 (98)	47 (94)	46 (100)	44 (96)					50/51 (98)	48/51 (94)
≥50% ƒT_>4xMIC_, n (%)	44 (88)	34 (68)	37 (80)	30 (65)					41/51 (80)	33/51 (65)
100% ƒT_>MIC_, n (%)	43 (91)	36 (77)	37 (88)	32 (76)	24 (86)	20 (71)	12 (92)	12 (92)	44/51 (86)	36/51 (71)
100% ƒT_>4xMIC_, n (%)	29 (62)	20 (43)	28 (67)	18 (43)	15 (54)	9 (32)	7 (54)	5 (38)	23/51 (45)	15/51 (29)
100% ƒT_>10xECOFF_, n (%)		7 (15)		6 (14)		3 (11)		2 (15)		9/51 (18)

% ƒT_>nxMIC_: percentage of dosing period in which the unbound concentration of cefazolin is n times above the MIC.

## Data Availability

The datasets used and/or analyzed in the current study are available from the corresponding author upon reasonable request.

## Supplementary Materials

The following supporting information can be downloaded at: https://www.mdpi.com/article/10.3390/antibiotics13100928/s1, Figure S1: Flow diagram. Figure S2: Correlation of serum albumin (left) and eGFR (right) with the unbound cefazolin fraction. CZO: cefazolin. The dashed line represents the published unbound cefazolin fraction (20%) in healthy volunteers. Figure S3: Comparison of differences between measured and estimated mid-dose and trough unbound serum cefazolin vs serum albumin concentration measured simultaneously. CZO: cefazolin. Figure S4: Goodness-of-fit plots for the final joint model for unbound concentrations. IWRES: individual weighted residuals. Figure S5: Goodness-of-fit plots for the final joint model for total concentration. Figure S6: Study design. Table S1: Patient characteristics and univariate analysis between patients who attained the primary endpoint of 100% *f*T_>1xMIC_ and those who did not. Table S2: Population parameter estimates. CL: clearance; V: volume of distribution; Bmax: maximum binding capacity; NS: non-saturable constant; kd: dissociation constant; GFR: eGFR-GC; GFR_mean_: 80.1634 (weighted mean GFR-GC); GFR_CL_: exponent for the allometrically scaled estimated glomerular filtration rate (eGFR-GC) on clearance; WeightV; exponent for the allometrically scaled weight on volume of distribution; Albumin_mean_: 24.0844 (weighted mean albumin); Albumin_NS_: exponent for the allometrically scaled albumin on kd; CI: confidence interval; RSE: relative standard error [%RSE = 100 × (standard error/parameter estimate)]. Table S3. Most important PopPK model building steps. Cl: clearance; CMT: compartments; OBV: objective function value (log likelihood value); Vd: volume of distribution.
