# Supplementary material for: Target Attainment and Population Pharmacokinetics of Cefazolin in Patients with Invasive Staphylococcus aureus Infections: A Prospective Cohort Study
Source: Antibiotics (Basel). 2024 Sep 29;13(10):928. doi: 10.3390/antibiotics13100928 (PMC11504871; doi:10.3390/antibiotics13100928)
Supplement: Supplementary file 1 [file antibiotics-13-00928-s001.zip › antibiotics-3182376-supplementary.pdf]

Supplement

Figure S1. Flow diagram. COVID-19: coronavirus disease 2019.

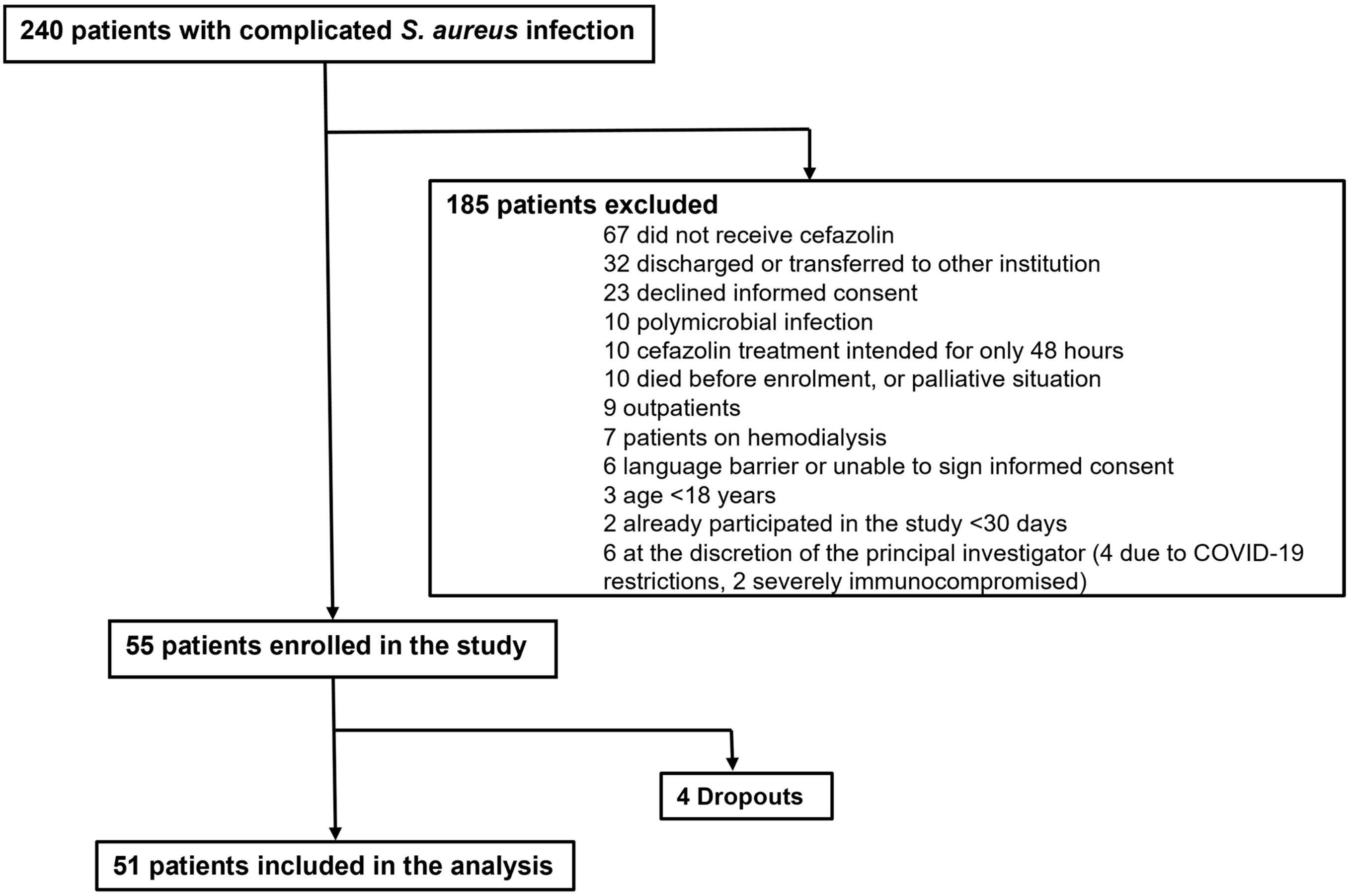

Figure S2. Correlation of serum albumin (left) and eGFR (right) with the unbound cefazolin fraction. Albumin and eGFR were determined from the same blood sample as cefazolin. CZO: cefazolin. The dashed line represents the published unbound cefazolin fraction (20%) in healthy volunteers.

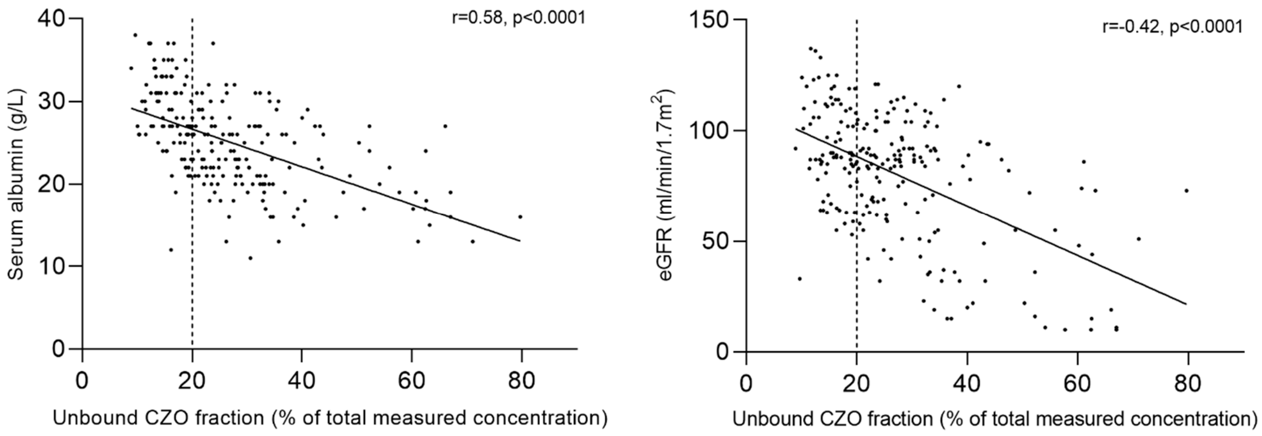

Figure S3. Comparison of difference between measured and estimated mid-dose and trough unbound serum cefazolin vs serum albumin concentration measured simultaneously. CZO: cefazolin.

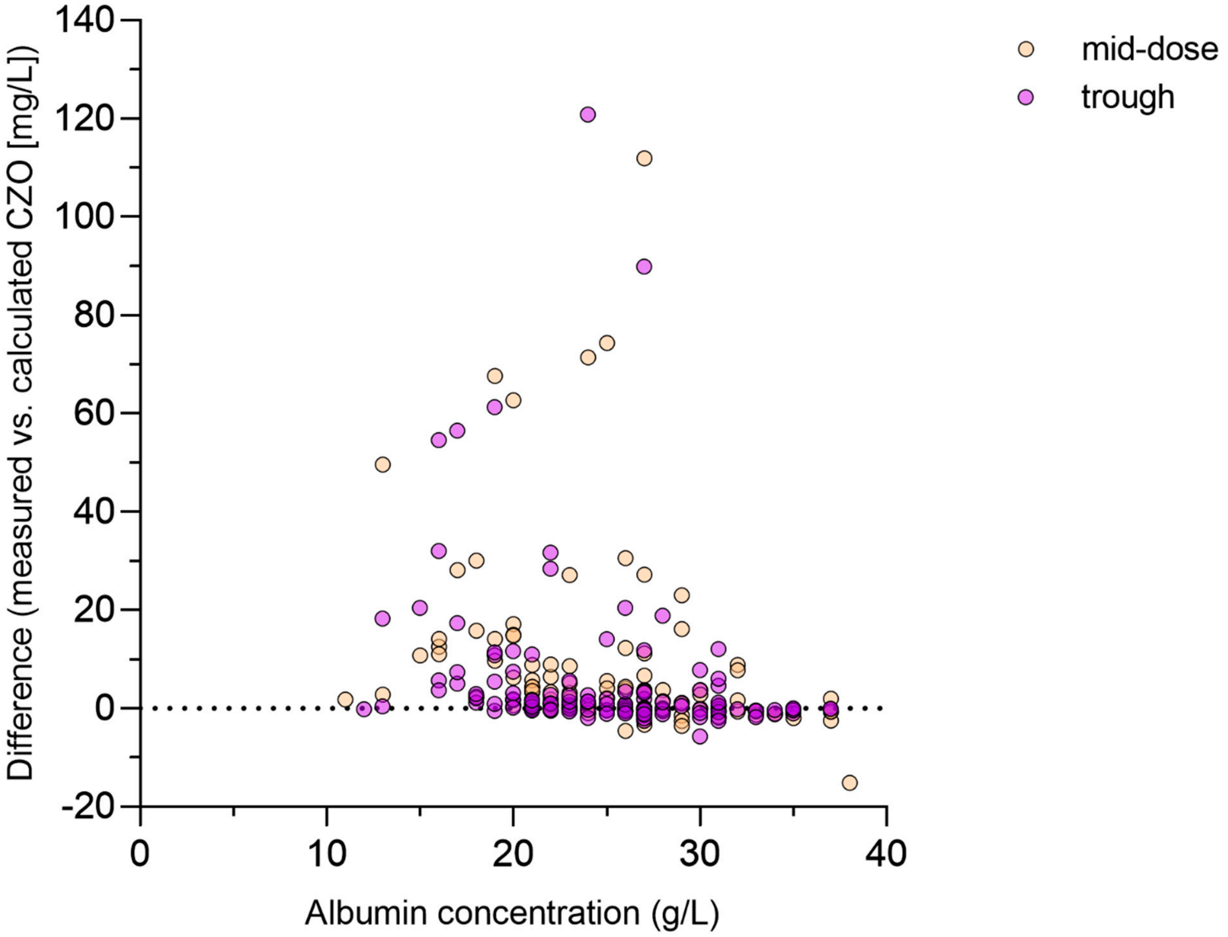

Figure S4. Goodness-of-fit plots for the final joint model for unbound concentration. IWRES: Individual weighted residuals

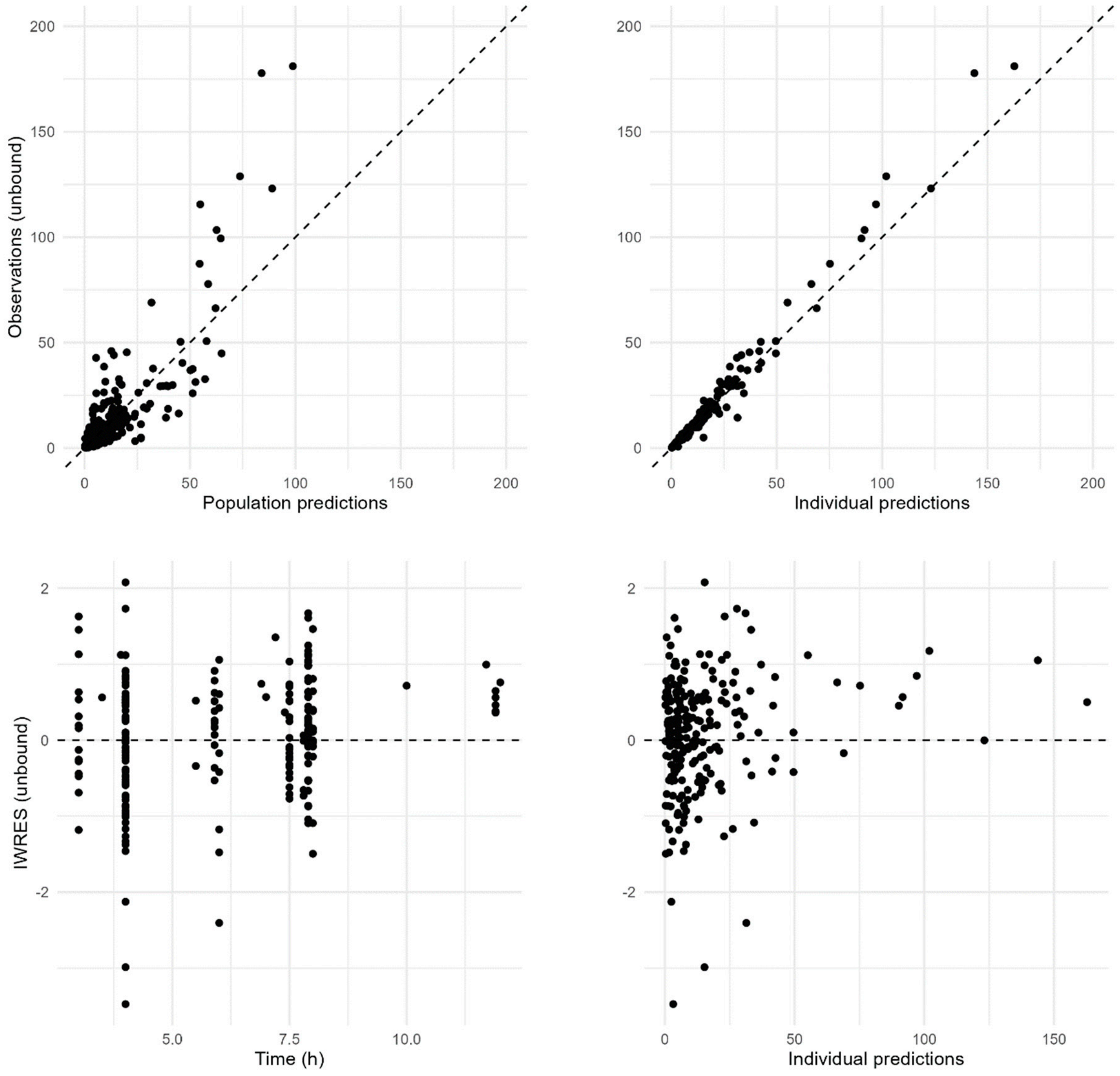

Figure S5. Goodness-of-fit plots for the final joint model for total concentration. IWRES: Individual weighted residuals

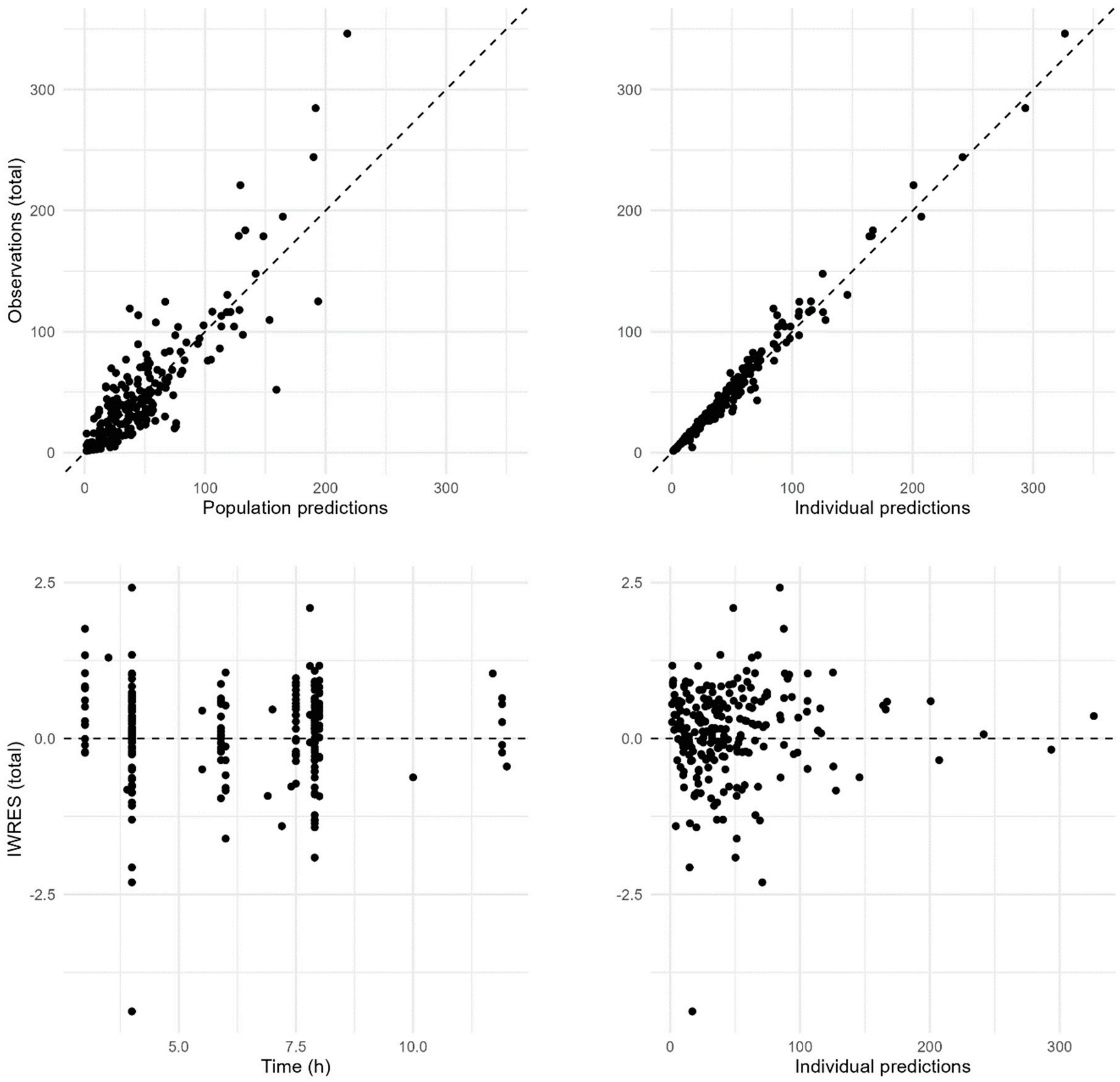

Figure S6. Study design

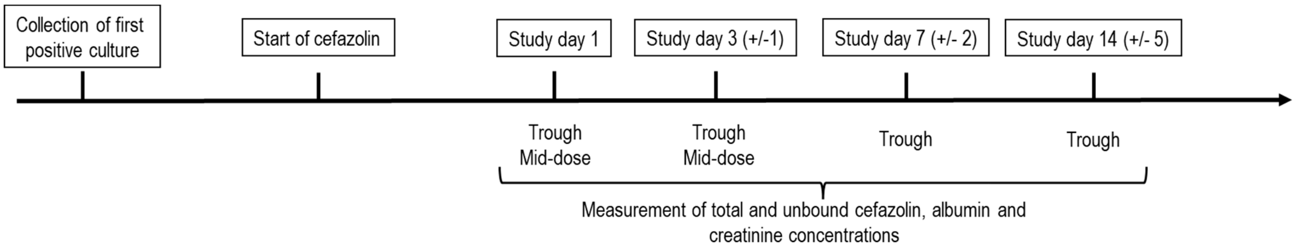

Table S1. Patient characteristics and univariate analysis between patients who attained the primary endpoint of 100% $fT_{>1xMIC}$ and those who did not.

	**Target non-attainment (N=7)**	**Target attainment (N=44)**	**Total (N=51)**	**p-value**
Age (years)	42.4 (33.9-52.7)	76.8 (62.6-81.9)	74.1 (57.3-81.1)	**< 0.001**
Gender, female (%)	2 (29)	11 (25)	13 (26)	1
BMI (kg/m^2)	31.6 (26.9-32.8)	24.4 (21.9-28.7)	24.5 (22.4-29.2)	0.138
Charlson Comorbidity Score	1 (0-1)	5 (3-8)	5 (2-7)	**< 0.001**
SOFA Score at onset of infection	1 (0.5-1)	2 (1-4)	1 (1-3)	**0.036**
Focus of infection (%)				
catheter and/or foreign material	3 (43)	10 (23)	13 (26)	
intra-abdominal	1 (14)	0 (0)	1 (2)	
respiratory tract	0 (0)	2 (5)	2 (4)	
skin soft tissue	2 (29)	7 (16)	9 (18)	
urinary tract	0 (0)	0 (0)	0 (0)	
endocarditis	0 (0)	10 (23)	10 (20)	
other	0 (0)	6 (14)	6 (12)	
osteomyelitis or arthritis	1 (14)	9 (21)	10 (20)	
Leucocytes (10^9/L) at first drug measurement	8.2 (6.2-14.3)	8.2 (6.5-11.5)	8.2 (6.4-11.8)	0.891
Platelets (10^9/L) at first drug measurement	443 (276-517)	262 (169-363)	277 (171-406)	0.1
CRP (mg/L) at first drug measurement	41 (38-113)	98 (43-175)	97 (39-172)	0.502
eGFR (ml/min/1.7m^2) at first drug measurement	117 (114-123)	87 (68-89)	87 (70-102)	**0.002**
Albumin (g/L) at first drug measurement	30 (27-34)	25 (21-27)	25 (22-27)	**0.037**
ICU admission (%)	1 (14)	9 (21)	10 (20)	0.703
30-day mortality (%)	0 (0)	3 (7)	3 (6)	0.187

P-values in bold are statistically significant at a level of <0.05.

Table S2. Population parameter estimates. CL: clearance; V: volume of distribution; Bmax: maximum binding capacity; NS: non-saturable constant; kd: dissociation constant; GFR: eGFR-CG; GFR_{mean}: 80.1634 (weighted mean GFR-CG); GFR_{CL}: exponent for the allometrically scaled estimated glomerular filtration rate (eGFR-CG) on clearance; $Weight_{V;}$ exponent for the allometrically scaled weight on volume of distribution; $Albumin_{mean}$: 24.0844 (weighted mean albumin); $Albumin_{NS:}$ exponent for the allometrically scaled albumin on kd; CI: confidence interval; RSE: relative standard error [%RSE = 100 * (standard error/parameter estimate)]

	Final joint model	Bootstrap analysis (n=1'000)	
	Estimate [%RSE]	Median	95% CI
**Fixed effects**			
CL_{pop}, l h^{-1}	15.6 [3.8]	16.2	13.4-19.1
V_{pop}, l	67.2 [7.3]	71.2	55.7-90.1
$Bmax_{pop}$	55.2 [10.7]	54.1	33.5-80.1
kd_{pop}	12.7 [10.1]	12.9	7.1-21.2
NS_{pop}	0.5 [18.6]	0.6	0.4-1.0
GFR_{CL}	1.2 [5.0]	1.2	0.9-1.4
$Weight_{V}$ (fixed)	1.0	n/a	n/a
$Albumin_{NS}$	2.4 [12.0]	2.8	1.8-3.9
**Inter-individual variability (IIV)**			
V_{IIV}	0.4 [15.6]	0.4	0.2-0.6
$Bmax_{IIV}$	0.2 [21.0]	0.2	0.1-0.3
**Inter-occasional variability (IOV)**			
CL_{IOV}	0.4 [7.12]	0.4	0.3-0.5
NS_{IOV}	0.2 [31.0]	0.2	0.1-0.3
**Residual error**			
Proportional (b)			
Total	0.2 [8.33]	0.2	0.1-0.2
Unbound	0.2 [7.79]	0.2	0.2-0.3
**Covariate Relationships**			
CL	$CL_{pop} * \left(\frac{GFR}{GFR_{mean}}\right)^{GFR_{CL}}$		
V	$V_{pop} * \left(\frac{Weight}{70}\right)^{Weight_{V}}$		
NS	$NS_{pop} * \left(\frac{Albumin}{Albumin_{mean}}\right)^{Albumin_{NS}}$		

Table S3. Most important PopPK model building steps. Cl: clearance; CMT: compartments; OBV: objective function value (log likelihood value); Vd: volume of distribution

CMT	Covariates	Error	OFV
Total concentration			
1	None	Combined 1	1869.37
1	GFR-CG on Cl	Combined 1	1783.85
1	GFR-CG on Cl<br>Albumin on Vd	Combined 1	1782.6
1	GFR-CG and albumin on Cl	Combined 1	1784.24
1	GFR-CG on Cl<br>Weight on Vd	Combined 1	1770.78
1	GFR-CG on Cl	Proportional	1790.17
1	GFR-CG on Cl<br>Weight on Vd	Proportional	1772.14
2	None	Combined1	1835.13
2	GFR-CG on Cl	Combined 1	1811.63
3	None	Combined 1	1901.32
3	GFR-CG on Cl	Combined 1	1814.25
Unbound concentration			
1	None	Combined 1	1349.80
1	GFR-CG on Cl	Combined 1	1285.05
1	GFR-CG on Cl<br>Weight on Vd	Combined 1	1268.66
1	GFR-CG on Cl<br>Albumin on Vd	Combined 1	1287.43
1	GFR-CG and albumin on Cl	Combined 1	1280.08
1	GFR-CG and albumin on Cl<br>Weight on Vd	Combined 1	1259.22
2	None	Combined 1	1290.35
2	GFR-CG on Cl	Combined 1	1230.69
2	GFR-CG on Cl<br>Albumin on Q	Combined 1	1241.01
3	None	Combined 1	1311.48
3	GFR-CG on Cl	Combined 1	1268.63
**Total and unbound concentration**			
Protein binding fraction unbound (fu)			
1	None	Combined 1	2917.87
1	GFR-CG on Cl	Combined 1	2833.13
1	GFR-EPI on Cl	Combined 1	2845.04

CMT	Covariates	Error	OFV
1	GFR-CG on Cl<br>Albumin on fu	Combined 1	2818.30
1	GFR-CG on Cl<br>Albumin on fu<br>Weight on Vd	Combined 1	2800.22
1	GFR-CG on Cl<br>Albumin on fu<br>Weight on Vd	Proportional	2801.47
Protein binding linear with dissociation constant (kd)			
1	None	Combined 1	2972.5
1	GFR-CG on Cl	Combined 1	2908.23
1	GFR-CG on Cl<br>Albumin on kd	Combined 1	2884.38
1	GFR-CG on Cl<br>Albumin on kd<br>Weight on Vd	Combined 1	2866.33
1	GFR-CG on Cl<br>Albumin on kd<br>Weight on Vd	Proportional	2917.21
Protein binding non-linear (kd) and saturable (Bmax)			
1	None	Combined 1	2917.21
1	GFR-CG on Cl	Combined 1	2853.68
1	GFR-CG on Cl<br>Albumin on Bmax	Combined 1	2818.6
1	GFR-CG on Cl<br>Albumin on kd	Combined 1	2824.95
1	GFR-CG on Cl<br>Albumin on kd<br>Weight on Vd	Combined 1	2809.32
Protein binding non-linear (kd), saturable (Bmax) and unsaturable (NS)			
1	None	Combined 1	2922.11
1	GFR-CG on Cl	Combined 1	2865.13
1	GFR-CG on Cl<br>Albumin on kd	Combined 1	2854.78
1	GFR-CG on Cl<br>Albumin on NS<br>Weight on Vd	Combined 1	2816.93

**CMT**	**Covariates**	**Error**	**OFV**
1	GFR-CG on Cl<br>Albumin on kd<br>Weight on Vd	Proportional	2871.64
